# Connexin-43-Mediated Gap Junction Coupling Between Adipocytes Regulates Norepinephrine-Induced Ca^2+^ Responses in Perivascular Adipose Tissue

**DOI:** 10.3390/cells15100906

**Published:** 2026-05-15

**Authors:** Ae Ra Kim, Julia Jamka, William F. Jackson, Emma D. Flood, Jonathon L. McClain, Brian D. Gulbransen

**Affiliations:** 1 Department of Physiology, College of Natural Science, Michigan State University, 567 Wilson Road, East Lansing, MI 48824, USA; kimaera@msu.edu (A.R.K.); mcclai11@msu.edu (J.L.M.); 2 Department of Pharmacology and Toxicology, College of Osteopathic Medicine, Michigan State University, East Lansing, MI 48824, USA

**Keywords:** PVAT, adipose, fat, gap junctions, connexin-43, adrenergic, vascular, calcium

## Abstract

**Highlights:**

**What are the main findings?**

- Adipocytes in perivascular adipose tissue (PVAT) express gap junctions composed of connexin-43.
- Gap junction coupling between PVAT adipocytes regulates responses to norepinephrine.

**What are the implications of the main finding?**

- Gap junction coupling between PVAT adipocytes provides a potential explanation for how signals from limited sympathetic innervation are distributed throughout PVAT depots.
- While adipocyte gap junction coupling could contribute to the mechanism by which PVAT controls blood vessel contractility, the functional relevance of this mechanism remains unclear because adipocyte-specific connexin-43 knockout mice showed no vascular phenotype and upregulated connexin 26 gene expression, which may indicate potential compensatory mechanisms.

**Abstract:**

Anticontractile factors secreted by perivascular adipose tissue (PVAT) play an important role in regulating vascular tone. This process is driven by the neurotransmitter norepinephrine (NE), but recent data show that adrenergic innervation in PVAT is sparse. How limited innervation might initiate broad responses through PVAT depots remains unknown. Here, we used Ca^2+^ imaging with genetically encoded sensors, selective drugs, immunolabeling and a conditional ablation model to test the hypothesis that gap junction coupling among PVAT adipocytes contributes to how signals initiated by NE are distributed through PVAT depots. Despite exhibiting differing sensitivities to NE, adipocytes in aortic and mesenteric PVAT and in white adipose tissue displayed robust expression of the gap junction protein connexin-43 (Cx43). Blocking gap junction coupling with the drug carbenoxolone (Cbx) limited NE-evoked Ca^2+^ responses among adipocytes, while blocking Cx43 hemichannels with the mimetic peptide 43Gap26 had no significant effect. Fluorescence recovery after photobleaching (FRAP) in mPVAT was decreased in the presence of Cbx, suggesting impaired gap junction communication. Wire myography recordings of mesenteric arteries showed that the EC_50_ for NE was higher in samples with intact PVAT than those without; however, this effect was not significantly different in samples from mice that lacked Cx43 in adipocytes. Analysis of multiple connexins showed that adipocytes upregulate Cx26 gene expression when Cx43 is deleted. These observations support the conclusion that Cx43-mediated gap junction coupling among PVAT adipocytes contributes to distributing signals initiated by NE; however, how this mechanism contributes to regulating vessel constriction remains unclear. This, and how potential compensatory mechanisms are enacted in adipocytes lacking Cx43, should be addressed in future work.

## 1. Introduction

Cardiovascular diseases are the leading cause of death globally and contribute to major personal and societal costs [1]. Hypertension is one of the most common contributors to cardiovascular disease and affects approximately half of Americans [2]. While processes involved in the pathogenesis of hypertension are often considered to work through inside-out signaling mechanisms that produce blood vessel dysfunction, an emerging concept is that outside-in signaling also plays a significant role in vascular pathophysiology [3]. Much of this outside-in signaling is thought to involve a unique type of adipose tissue called perivascular adipose tissue (PVAT) that surrounds most blood vessels and releases diffusible factors that regulate local vascular reactivity and inflammatory status [4]. PVAT exhibits unique qualities depending on its anatomical location that reflect differences in adipocyte progenitors, differentiation, cellular morphology and cellular composition. Adipocytes are the most abundant cell type, but PVAT also contains immune cells, endothelial cells, fibroblasts and adipocyte stem/progenitor cells. Changes in the cellular composition and/or function of PVAT is now considered an important contributor to pathophysiological changes that occur in cardiovascular diseases, diabetes and obesity [5,6,7,8,9,10,11]. Yet how PVAT exerts beneficial and deleterious effects on the vasculature remains poorly understood.

Vasoactive actions of PVAT adipocytes are mediated, in part, by several secreted anticontractile factors that include adiponectin, leptin, nitric oxide (NO), hydrogen sulfide (H_2_S), and chemerin, among others [12]. Norepinephrine (NE) appears to play an important role in promoting the release of these factors from adipocytes and, hence, stimulating the anticontractile nature of PVAT [13,14]. This has raised the possibility that sympathetic innervation may play dual roles in regulating the vasculature through direct actions that promote vasoconstriction and indirect actions through innervation of PVAT that promote vasodilation. Despite this, emerging data show that adrenergic innervation of PVAT is extremely limited [15,16]. How such sparse innervation could regulate the activity of many adipocytes within PVAT depots remains unknown.

A possible explanation for how limited innervation could produce broader effects through PVAT has emerged from work on mechanisms underlying beiging in white adipose tissue. Here, responses to minimal sympathetic innervation are propagated between adipocytes through cell–cell signaling involving gap junction-coupled networks composed of connexin-43 (Cx43) [17]. Gap junctions provide direct and selective channels between neighboring cells and allow the passage of molecules smaller than 1000 daltons including inorganic ions such as Ca^2+^ and Na^+^, second messengers such as cAMP and inositol 1,4,5-trisphosphate, small RNAs and certain metabolites [18,19]. Gap junctions and hemichannels, unpaired connexons that connect the intracellular space with the extracellular environment, are regulated by several factors that include transmembrane voltage [20], cell redox potential [21], phosphorylation [22,23], and cations [24,25] such as intracellular Ca^2+^ [26,27]. Gap junctions play a vital role in regulating vascular function [28,29] and are implicated in mechanisms involved in the pathophysiology of diabetes and hypertension [30,31,32,33].

Given the important roles of gap junction coupling in white adipose tissue and in vascular function, we reasoned that similar mechanisms may explain how PVAT adipocytes propagate signals initiated by limited sympathetic innervation. We tested potential contributions of gap junction coupling to PVAT adipocyte responses driven by NE in calcium (Ca^2+^) imaging experiments and conducted immunolabeling experiments to localize Cx43 in PVAT. Functional assessment of gap junction activity was performed using fluorescence recovery after photobleaching (FRAP), and roles of adipocyte Cx43 in controlling small resistance artery contractile properties were evaluated in adipocyte-specific Cx43 (Adipo^∆Cx43^) knockout mice. Together, these results provide valuable insight into the significance of gap junction coupling in PVAT as a pathway of cellular communication.

## 2. Results

### 2.1. Norepinephrine (NE) Stimulates Ca^2+^ Responses Among Adipocytes in Perivascular Adipose Tissue (PVAT)

**Figure 1:**
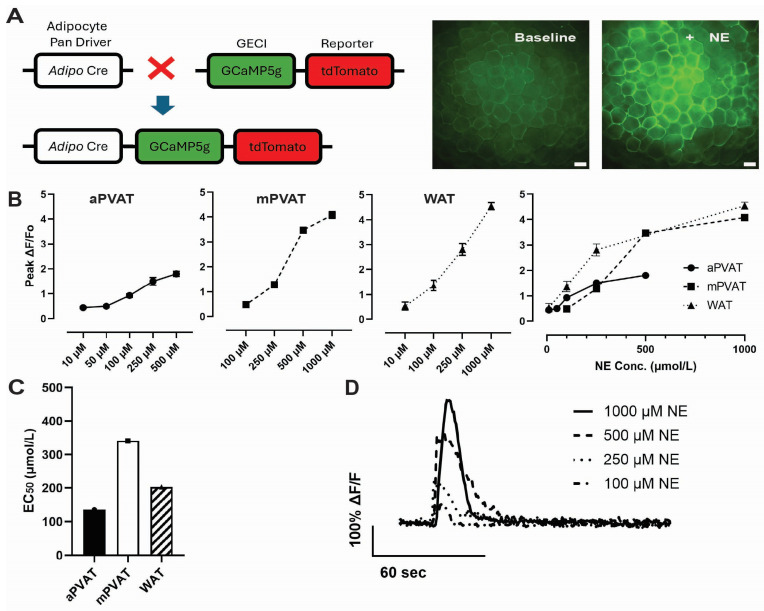
Norepinephrine (NE) stimulates Ca^2+^ responses among adipocytes in perivascular adipose tissue (PVAT). (**A**) Model depicting *Adipoq^Cre+^;GCaMPtdt^f/Wt^* mice expressing the genetically encoded calcium indicator, GCaMP5g, and reporter protein, tdTomato, under control of the adipocyte-selective driver, adiponectin. (**B**) Quantification of adipocyte Ca^2+^ response amplitude (peak ∆F/Fo) following focal stimulation with NE in aortic perivascular adipose tissue (aPVAT), mesenteric perivascular adipose tissue (mPVAT) and white adipose tissue (WAT) (n = 30–280 from 3 animals per experimental group). Data are presented as mean ± SEM. (**C**) EC_50_ values calculated from NE concentration–response curves. (**D**) Representative traces of NE-evoked Ca^2+^ responses in mPVAT. Scale bar = 50 µm.

Given the critical role of PVAT in vascular homeostasis and the emerging evidence that NE stimulates the release of anticontractile factors from PVAT [6,7,9], we began by assessing the sensitivity of different PVAT depots to NE using Ca^2+^ imaging. In prior work, we developed *Adipoq^cre+^;GCaMP5g-tdT^f/WT^* mice (Figure 1A) as a tool to visualize and quantify NE-induced Ca^2+^ propagation in PVAT adipocytes and showed that exposing samples of PVAT to NE evokes robust Ca^2+^ responses among adipocytes that are mediated by a1a, b2 and b3 adrenergic receptors and the subsequent release of Ca^2+^ from intracellular stores, with depot-specific differences [15,34]. However, whether different adipose depots display differing sensitivities to NE has remained unclear. We addressed this issue by conducting ex vivo live-cell Ca^2+^ imaging in samples of aortic PVAT (aPVAT), mesenteric PVAT (mPVAT) and white adipose tissue (WAT) and challenging the adipocytes with NE concentrations ranging from 10 to 1000 µmol/L.

Adipocytes in aPVAT, mPVAT and WAT were responsive to NE, but sensitivities and maximal responses evoked differed (Figure 1B). Low-amplitude Ca^2+^ transients were observable in aPVAT in response to NE concentrations as low as 10 µmol/L and NE-driven Ca^2+^ responses saturated around 250 µmol/L. In contrast, WAT adipocytes exhibited a larger dynamic range and maintained responses up to 1000 µmol/L. mPVAT adipocytes exhibited a response profile that was similar to those in WAT, which agrees with the concept of a common phenotype between adipocytes in these depots [35]. However, mPVAT adipocytes were less sensitive to NE than those in WAT and responses to NE below 100 μmol/L were not observed, which is supported by half-maximal effective concentration (EC_50_) values 135 µmol/L, 341.1 µmol/L and 202.5 µmol/L at aPVAT, mPVAT and WAT, respectively (Figure 1C). Upon NE stimulation, adipocytes in aPVAT produced Ca^2+^ responses with average amplitudes of 0.45 ± 0.04, 0.49 ± 0.06, 0.88 ± 0.07, 1.5 ± 0.15, and 1.8 ± 0.10 ΔF/F_0_ at 10, 50, 100, 250, and 500 μmol/L NE, respectively. In contrast, mPVAT exhibited responses to NE that were 0.48 ± 0.07, 1.28 ± 0.11, 3.47 ± 0.09, and 4.08 ± 0.13 ΔF/F_0_ at 100, 250, 500 and 1000 μmol/L NE. Adipocytes in WAT exhibited responses of 0.53 ± 0.17, 1.36 ± 0.20, 2.81 ± 0.24, and 4.55 ± 0.15 ΔF/F_0_ at 10, 100, 250 and 1000 μmol/L NE, respectively. Taken together, these data demonstrate that NE stimulation elicits robust, concentration-dependent Ca^2+^ responses in adipocytes from aPVAT, mPVAT and WAT (Figure 1B,D). The distinct NE response profiles observed among aPVAT, mPVAT and WAT suggest that PVATs have different sensitivities to NE stimulation that are dependent on their anatomical location and tissue composition [36,37,38].

### 2.2. Expression of Connexin-43 in PVAT from WT and Adipo^∆Cx43^ KO Mice

To determine whether the propagation of Ca^2+^ responses between adipocytes could be functionally regulated by connexin hemichannels, we began assessing the presence of connexin-43 (Cx43) in PVAT using immunofluorescence staining. Cx43 is one of the major connexin subtypes that plays an important role in gap junction communication. Immunolabeling was performed with antibodies against Cx43 and the membrane marker Caveolin-1 [39,40] in WT mice and adipocyte-specific Cx43^(f/f)^ knockout (Adipo^∆Cx43^ KO) mice.

**Figure 2:**
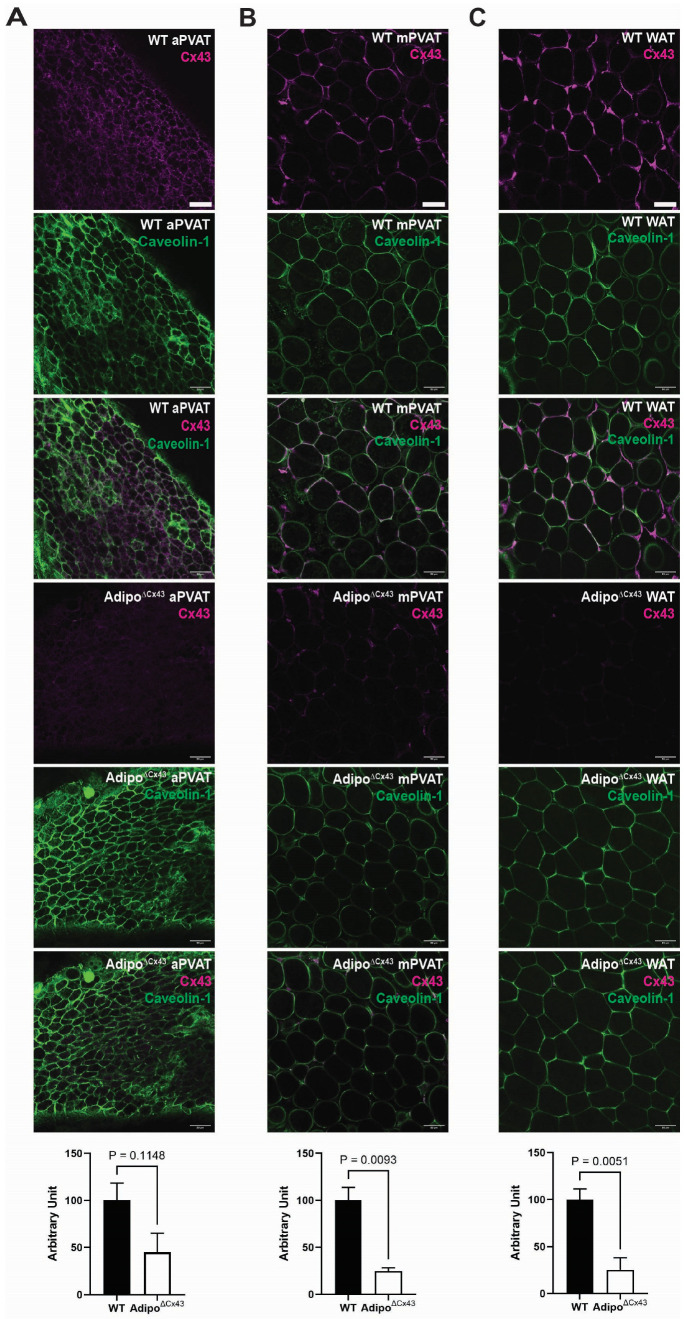
Expression of connexin-43 (Cx43) in PVAT from WT and Adipo^∆Cx43^ KO mice. Immunofluorescent labeling and quantification of Cx43 (magenta) and Caveolin-1 (green) in aPVAT (**A**), mPVAT (**B**), WAT (**C**). Images are representative of labeling in n = 3–4 mice. Scale bar = 50 µm.

As shown in Figure 2, Cx43 protein expression was distributed around the nucleus and in large foci at cell–cell borders, corresponding to gap junctions between adjacent cells. No notable alterations or patterns of Cx43 expression were observed between different PVATs. Control experiments showed a loss of Cx43 labeling in samples of PVAT from Adipo^∆Cx43^ KO mice, confirming the specificity of the antibody.

### 2.3. Cx43 Gap Junctions, but Not Hemichannels, Modulate NE-Evoked Ca^2+^ Responses in PVAT

**Figure 3:**
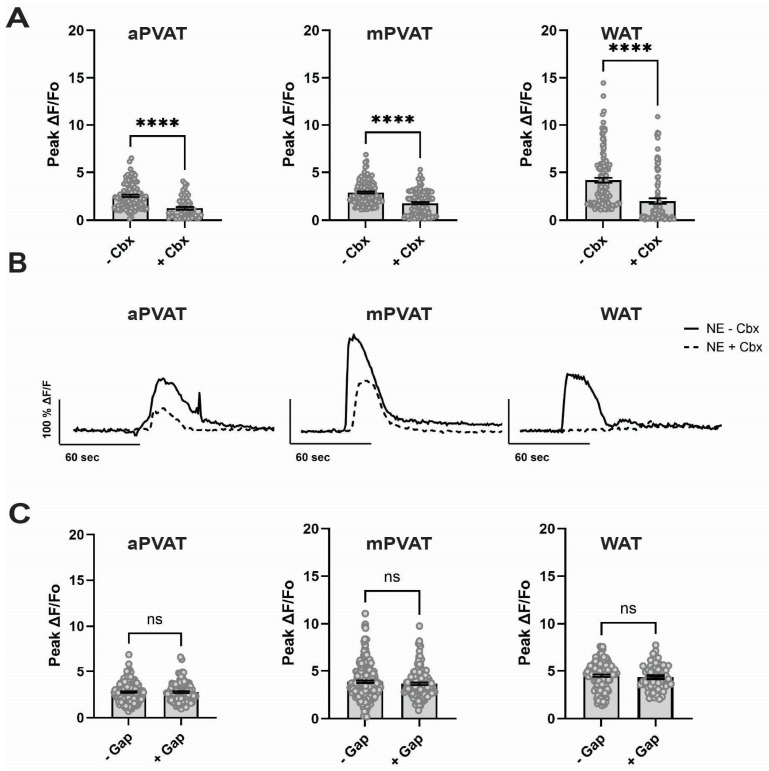
Gap junctions, but not Cx43 hemichannels, modulate NE-evoked Ca^2+^ responses in PVAT. (**A**) Effect of carbenoxolone (Cbx; 50 µmol/L) on the amplitude of adipocyte Ca^2+^ responses to NE in aPVAT, mPVAT, and WAT. NE was applied at 100 µmol/L in aPVAT, 500 µmol/L in mPVAT, and 1000 µmol/L in WAT. These concentrations were determined based on differing sensitivities to NE calculated in earlier experiments. (**B**) Representative traces of NE-evoked Ca^2+^ responses in adipocytes in the presence or absence of Cbx. (**C**) Effect of blocking Cx43 hemichannels with the mimic peptide 43Gap26 (100 µmol/L) on the amplitude of adipocyte Ca^2+^ responses to NE in aPVAT, mPVAT, and WAT. Data are presented as mean ± SEM, n = 62–161 from 3 animals per experimental group, **** *p* < 0.0001, two-tailed *t* test.

Cx43 can form gap junctions that provide a direct route of cell–cell coupling or can form hemichannels that provide a route to release diffusible factors into the extracellular space. Either of these two mechanisms could affect how adipocytes in PVAT distribute responses to NE among neighboring cells. To differentiate between roles of gap junctions and hemichannels in NE-driven Ca^2+^ responses in PVAT adipocytes, we conducted experiments in which we blocked gap junctions using the broad antagonist carbenoxolone disodium salt (Sigma, C4790, Cbx) or blocked hemichannels using the Cx43 mimetic peptide 43Gap26 (Anaspec Inc. 62644). Cbx significantly decreased NE-driven Ca^2+^ responses by 52% in aPVAT (1.56 ± 0.10 ΔF/F_0_ to 0.82 ± 0.09 ΔF/F_0_; *p* < 0.0001, n = 62 cells, three mice), by 64% in mPVAT (2.46 ± 0.11 ΔF/F_0_ to 1.59 ± 0.09 ΔF/F_0_; *p* < 0.0001, n = 161 cells, three mice) and by 48% in WAT (3.68 ± 0.23 ΔF/F_0_ to 1.75 ± 0.22 ΔF/F_0_; *p* < 0.0001, n = 121 cells, three mice) (Figure 3A,B). In contrast, blocking Cx43 hemichannels with 43Gap26 had no significant effect on Ca^2+^ responses to NE in aPVAT (2.78 ± 0.08 ΔF/F_0_ to 2.78 ± 0.11 ΔF/F_0_ with 43Gap26, n = 98 cells, three mice), mPVAT (3.69 ± 0.12 ΔF/F_0_ to 3.67 ± 0.12 ΔF/F_0_ with 43Gap26, n = 145 cells, three mice), or WAT adipocytes (4.5 ± 0.12 ΔF/F_0_ to 4.34 ± 0.16 ΔF/F_0_ with 43Gap26, n = 67 cells, three mice) (Figure 3C). These data are consistent with the conclusion that gap junction coupling among PVAT adipocytes plays an important role in propagating Ca^2+^ signals among adipocytes while hemichannels are either not present or play a minor role.

### 2.4. Gap Junctional Communication in mPVAT

The preceding antagonist data suggest that Cx43 contributes to gap junctional coupling among adipocytes. However, to rule out potential off target effects of Cbx, we conducted more direct assays of functional coupling between mPVAT adipocytes using FRAP [41,42,43,44]. In these experiments, adipocytes were loaded with Calcein AM, which diffuses through gap junctions. Calcein AM fluorescence was photobleached in single adipocytes in the presence or absence of Cbx, and recovery of fluorescence mediated by diffusion from adjacent cells was monitored over 10 min.

**Figure 4:**
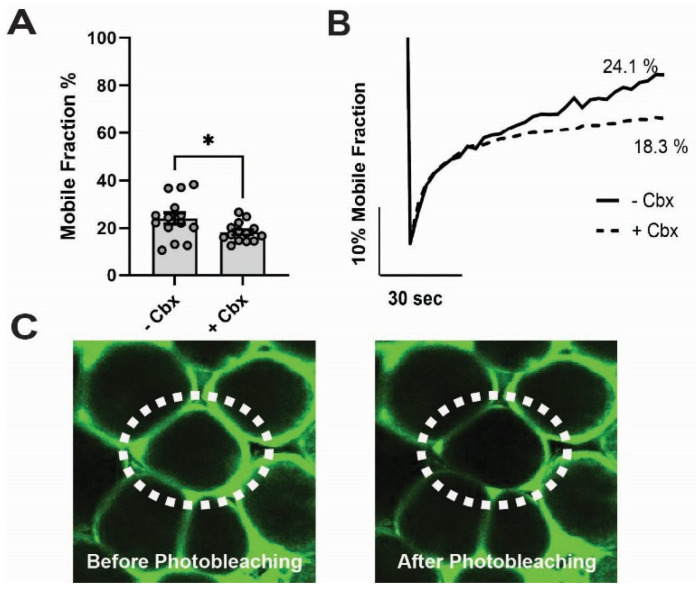
The gap junction blocker carbenoxolone (Cbx) reduces dye transfer between adipocytes. (**A**) The mobile fraction percentage values in mPVAT treated with of 50 µmol/L Cbx or vehicle control (n = 14 from 5 animals, data are presented as mean ± SEM, * *p* = 0.0363, two-tailed *t* test). (**B**) Representative traces of Calcein AM fluorescence recovery after photobleaching in the presence or absence of Cbx. (**C**) Representative confocal photomicrographs of mPVAT adipocytes. Cell (circle) is shown before photobleaching (**left**) and immediately after photobleaching (**right**).

On average, normal fluorescence recovery in the absence of Cbx was 24.07 ± 2.36% (n = 14 cells, five mice). In the presence of Cbx, fluorescence recovery was significantly reduced to 18.33 ± 1.09% (*p* = 0.0036, n = 14 cells, five mice) (Figure 4A–C). These observations suggest that functional gap junctions are present between adipocytes and form a conduit of intercellular communication between neighboring adipocytes in mPVAT. In addition, given the anatomical location of mPVAT next to mesenteric resistance vessels and the function of gap junctions in transferring small molecules between neighboring cells, it is possible that responses evoked in adipocytes distant from vessels could influence mesenteric resistance vascular function through gap junction-coupled networks.

### 2.5. Effects of Adipocyte-Specific Cx43 Deletion on NE-Induced Mesenteric Resistance Artery Constriction

**Figure 5:**
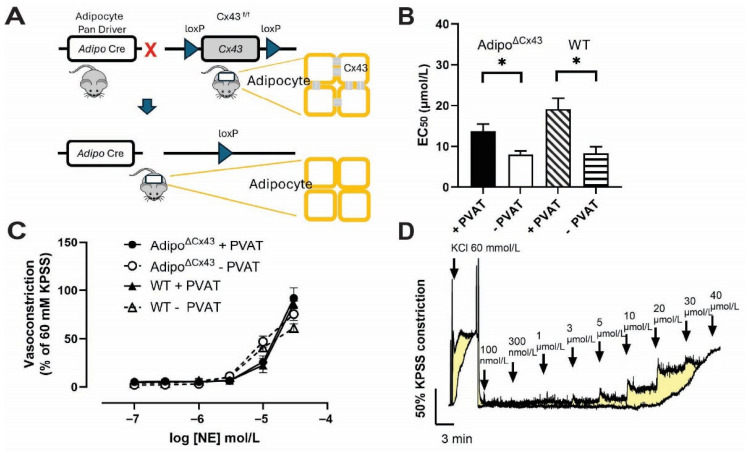
Effects of adipocyte-specific Cx43 deletion on NE-induced mesenteric resistance artery constriction. (**A**) Model depicting Adipo^∆Cx43^ KO mice with loxP sites flanking exon 2 of Cx43 (Cx43 ^f/f^) and Cre expression under control of the adipocyte driver, adiponectin. (**B**) EC_50_ values calculated from NE concentration–response curves normalized to maximum response from within each curve (n = 4–5 from a minimum of 4 animals per experimental group, data are presented as mean ± SEM, * *p* < 0.05, two-tailed *t* test, with PVAT vs. without PVAT). (**C**) The presence of PVAT significantly attenuated NE-induced constriction in both WT and Adipo^∆Cx43^ KO mice, indicating preservation of the anticontractile effect. (**D**) Representative constriction curves in response to potassium physiological salt solution (KPSS, including 60 mmol/L of KCl), and norepinephrine (0.1–40 µmol/L) in samples from WT mice with (bottom) and without (top) PVAT. The net beneficial anticontractile effect of PVAT on NE contraction is represented in yellow.

The release of anticontractile factors from PVAT is thought to be driven by NE and our data show that adipocyte responses to NE are regulated by gap junctions that are likely composed of Cx43. Therefore, we tested whether specifically deleting Cx43 from adipocytes would alter the effect of PVAT on vessel function. To do so, we created adipocyte-specific Cx43^(f/f)^ knockout (Adipo^∆Cx43^ KO) mice (Figure 5A) and carried out wire myography recordings on second- and third-order mesenteric resistance arteries isolated from both Adipo^∆Cx43^ KO mice and WT mice with or without PVAT [45,46].

In WT mice, the anticontractile effect of PVAT was evident by a rightward shift in the cumulative NE concentration–response curve (Figure 5B,C). The EC_50_ for NE was 19.13 ± 2.70 µmol/L in arteries with PVAT, compared to 8.30 ± 1.63 µmol/L in arteries without PVAT. Unexpectedly, PVAT retained its anticontractile effect in Adipo^∆Cx43^ KO mice and EC_50_ measurements with and without PVAT were comparable to those in WT (13.72 ± 1.75 µmol/L with PVAT and 8.00 ± 0.89 µmol/L without PVAT). These observations were surprising given the multiple other lines of data suggesting a significant role for Cx43 in PVAT responses to NE; however, the experimental conditions tested here in which NE was bath applied may preclude the need for gap junction coupling and limit a clear view of the role of adipocyte gap junction coupling in vessel function.

### 2.6. mRNA Expression of Connexin Isoforms in Wild Type and Adipo^∆Cx43^KO Mice

One possibility that could explain why deleting adipocyte Cx43 had no effect on vascular tone is that adipocytes compensate for the loss of Cx43 by upregulating other connexin isoforms that could mask the effects of deleting Cx43. To assess this possibility, we used real-time qPCR analysis to compare the expression of multiple connexin isoforms in mPVAT from Adipo^∆Cx43^ KO and WT animals. Primer sequences and amplicon sizes are listed in Table 1.

**Figure 6:**
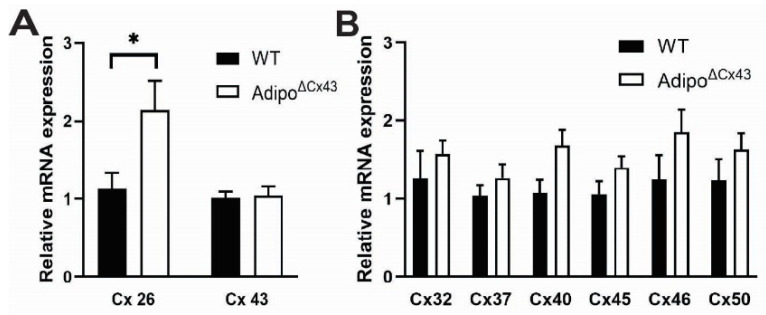
mRNA expression of connexin isoforms in wild type and Adipo^∆Cx43^ KO mice. (**A**) Relative mRNA expression of Cx26 and Cx43 (n = 6–8 animals per experimental group, data are presented as mean ± SEM, * *p* = 0.05, two-tailed *t* test). (**B**) Relative mRNA expression of Cx32, Cx37, Cx40, Cx45 and Cx50 (n = 6–8 animals per experimental group, data are presented as mean ± SEM). Values were normalized to the housekeeping gene RPS6, and relative Cx expression was quantified using the 2^−∆∆Ct^ method.

Despite exhibiting reduced Cx43 protein expression in adipocytes (Figure 2), Cx43 mRNA expression was comparable between samples of PVAT from Adipo^∆Cx43^ and WT mice. This difference is likely due to the fact that Cx43 is expressed by multiple cell types in PVAT in addition to adipocytes such as vascular smooth muscle. Interestingly, Cx26 mRNA increased by two-fold in Adipo^∆Cx43^ KO mice and ∆∆Ct values demonstrated statistically significant differences in WT (0.00 ± 0.84) and Adipo^∆Cx43^ KO (−0.95 ± 0.68) mice (n = 6–8, *p* = 0.048, two-tailed *t* test) (Figure 6A and Table 2). Expression of other connexin isoforms was not significantly different between Adipo^∆Cx43^ KO and WT mice, but exhibited several trends that could indicate subtle changes (Figure 6B). These data show that adipocytes exhibit a robust increase in Cx26 mRNA expression that may indicate compensation for the loss of Cx43 in the constitutive ablation model. It is possible that this upregulation of Cx26 masks the effects of deleting Cx43 on vessel function and contributes to the lack of effects observed.

## 3. Discussion

PVAT regulates vascular tone through mechanisms that involve factors released in response to NE. Here, we show that sensitivity to NE differs between PVAT depots and that responses to NE are influenced by cell–cell coupling among adipocytes. Cx43 is broadly expressed among PVAT adipocytes and blocking gap junctions impair their ability to respond to NE and effectively couple to neighboring cells. Interestingly, deleting Cx43 from adipocytes had no effect on the anticontractile effects of PVAT in vessel contractility experiments and this lack of effect could be due to multiple confounding factors including the experimental paradigm tested here and an upregulation of other connexins such as Cx26. While additional work is needed to understand these compensatory mechanisms and the role of Cx26 in more detail, our results show that gap junction coupling is an important mechanism that regulates how PVAT adipocytes function.

PVAT surrounds most blood vessels and was once considered to act primarily as connective tissue that supports vascular structure. However, contemporary concepts frame PVAT as a physiologically active and independent endocrine tissue that plays a critical role in maintaining vascular homeostasis. Multiple vasomodulatory factors are released by PVAT that exert anticontractile (adiponectin, nitric oxide, omentin, leptin, prostanoids, adipocyte-derived relaxing factor (ADRF)), pro-contractile (angiotensin-II, catecholamines, 5-HT, resistin, visfatin, chemerin, prostaglandin F_2α_), anti-inflammatory (IL-10, transforming growth factor (TGF)-β, prostacyclin) and pro-inflammatory (IL-1, IL-6, IL-8, TNF-α) effects [47,48,49,50]. Thus, the balance between these contrasting factors is critical for regulating vascular homeostasis.

The release of anticontractile factors from PVAT is thought to involve mechanisms that are enacted downstream of stimulation by NE [13,14]. Prior work showed that NE drives Ca^2+^ responses in PVAT adipocytes through mechanisms that involve α_1a_-, β_2_-, and β_3_-adrenergic receptors and downstream pathways that promote Ca^2+^ release from internal stores [15,34]. Importantly, elevations of intracellular Ca^2+^ have been implicated in the release of anticontractile factors [9,13,14,51]. In this study, we found that NE stimulation elicited robust adipocyte Ca^2+^ responses in all tissue types tested, although response profiles differed according to anatomical location. Adipocytes in aPVAT were sensitive to NE between 50 and 500 μmol/L, while those in mPVAT were less sensitive and responded in a range between 100 and 1000 μmol/L. WAT was more sensitive than mPVAT and maintained responsiveness at 1000 μmol/L of NE. It is well known that adipocytes in mPVAT and aPVAT are distinct and that mPVAT displays characteristics of white adipose tissue while aPVAT is more similar to brown adipose tissue [52,53,54]. This makes it likely that the differing sensitivities to NE could reflect their unique phenotypes and developmental origin. However, these differences may also suggest that different types of PVAT have specialized roles in vascular regulation and/or are designed to be recruited at differing times during sympathetic response. Additional work investigating how and why the various PVAT depots would be recruited differently by NE would be beneficial for understanding the physiological relevance of each tissue type.

Our immunolabeling data show that Cx43 is broadly expressed by adipocytes in aPVAT, mPVAT and WAT and Ca^2+^ imaging and FRAP experiments show that gap junction coupling plays an important role in regulating the breadth and magnitude of responses triggered by NE. Blocking gap junctions with the broad antagonist Cbx effectively reduced Ca^2+^ responses triggered by NE in every tissue. Likewise, data from FRAP experiments showed that normal dye transfer between adipocytes is impaired in the presence of Cbx. Both of these observations are consistent with the conclusion that PVAT adipocytes are extensively coupled by gap junctions and that gap junction coupling is important for distributing signals between adjacent adipocytes. Although Cbx is a broad gap junction blocker that is not selective for Cx43, these effects appear to be mainly mediated by traditional gap junctions and not hemichannels as the Cx43 hemichannel blocker 43Gap26 had no effect. Extensive gap junction coupling among PVAT adipocytes is interesting because this could explain the apparent mismatch between limited sympathetic innervation and the broad effects of NE on PVAT. Such a mechanism is consistent with what has been described previously in other adipose tissue depots [17]. In future work, it would be interesting to explore if defects in PVAT adipocyte gap junction coupling contribute to the proposed loss of the anticontractile nature of PVAT in conditions associated with hypertension.

As a first step in determining whether Cx43-mediated gap junction coupling plays a significant role in mechanisms by which PVAT regulates vascular tone, we examined mesenteric artery contractile responses to NE in mice lacking adipocyte Cx43 (Adipo^∆Cx43^ mice). Surprisingly, mesenteric resistance arteries displayed normal contractile responses to NE in mice lacking adipocyte Cx43. It is possible that this result suggests that adipocyte gap junction coupling is dispensable for mechanisms the control vascular tone, but this would be surprising given our data showing that gap junctions play a major role in adipocyte responses to NE and it is known that NE controls the release of PVAT anticontractile factors. It is also possible that technical limitations of the experimental setup limit observing a clear difference in response to NE. In these organ bath experiments, NE is bath applied and would conceivably have access to all adipocytes. This would likely limit the need for gap junction coupling as each adipocyte could be stimulated independently. While we cannot exclude this possibility, it is unlikely to be sufficient to fully explain the lack of effect because diffusion of NE into thick adipose tissue depots would be challenging and we also observed no obvious baseline differences between samples from WT and Adipo^∆Cx43^ KO mice. It is also possible that other connexin isoforms are upregulated to compensate for the functions of Cx43 when it is deleted. In support of this, prior studies in other tissues have observed compensation by connexin isoforms when Cx43 is deleted developmentally [55,56,57,58]. Our data show that Cx26 mRNA is significantly upregulated in PVAT from Adipo^∆Cx43^ KO mice. Cx26 and Cx43 share characteristics such as the secretory pathway, types of transport intermediates, turnover dynamics, and they may exist within the same gap junction plaques [59,60]. Therefore, it is possible that the upregulation of Cx26 could functionally compensate for the loss of Cx43 and maintain PVAT functions; however, additional work will be needed to determine whether functional compensation exists beyond changes in gene expression.

As indicated above, there are several limitations to this study. First, while the protein and gene expression data show that Cx43 is expressed by PVAT adipocytes, we cannot be entirely sure that the effects of Cbx are mediated solely by Cx43. Cbx is a broad gap junction inhibitor that also has potential effects on non-gap junction mechanisms. Based on the PCR data, multiple other connexin types are also expressed in PVAT and it remains unclear which of these are expressed by the adipocytes and which are expressed by other cell types that make up PVAT. Data from Ca^2+^ imaging, FRAP, immunolabeling and gene expression experiments are consistent with the conclusion that adipocytes are gap junction-coupled and that Cx43 is present, but additional work will be needed to determine the composition of the gap junctions and their specific nature. Second, the lack of functional effect in the adipocyte-specific Cx43 knockout model should be interpreted with caution for several reasons. As discussed above, a technical limitation of the experimental paradigm in wire myography recordings is that NE is bath applied and has the potential to influence many adipocytes without the need for gap junction coupling. This is certainly a confounding factor but is unlikely to fully account for the lack of effect given that not all adipocytes would be stimulated simultaneously due to limited diffusion through the thick tissue. Our data suggest that a potential alternate explanation is that adipocytes upregulate Cx26 when Cx43 is deleted developmentally. This observation is important for future work in this area and suggests that inducible, conditional knockouts or knockouts of several connexin genes will be needed to effectively reduce adipocyte gap junction coupling. If functional compensation by Cx26 or other connexin isoforms occurs in the knockout, it would contribute to reasons why no functional phenotype on vessel function was observed in this study. This knowledge would also be important for designing any studies going forward to avoid potential compensatory mechanisms. Lastly, although the gene expression data show a doubling of Cx26 mRNA expression, additional work is needed to determine if Cx26 forms functional gap junctions in PVAT adipocytes and truly compensates for the loss of Cx43. Although such compensation between connexins is common in other contexts, validating the role of Cx26 in PVAT will require additional work.

In conclusion, our data show that PVAT adipocytes express Cx43 and that gap junction coupling among the adipocytes regulates how adrenergic signals are distributed among the adipocytes in PVAT. These mechanisms are likely important for explaining how limited sympathetic innervation exerts broad effects through PVAT depots and possibly contributes to mechanisms that control how PVAT releases anticontractile factors. Given that defects in PVAT’s anticontractile mechanisms have received increased attention as potential contributors to hypertension, additional studies that test how these mechanisms are altered in disease states could produce important new targets to improve cardiovascular health.

## 4. Materials and Methods

### 4.1. Animals

Male and female mice aged 13–22 weeks old that expressed the genetically encoded Ca^2+^ indicator (GECI) GCaMP5g and the tdTomato reporter (tdT) in adipocytes were generated by crossing B6;*129s6-Polr2a^Tm1(CAG-GCaMP5g-tdTomato)Tvrd^*/J mice (Jackson Laboratory, Bar Harbor, ME, USA; Stock No. 024477; RRID: IMSR_JAX:024477) with B6.*FVB-Tg(Adipoq-cre)1Evdr*/J mice (Jackson Labotatory, Stock No. 028020; RRID: IMSR_JAX:028020). Offspring, *Adipoq^cre^;GCaMP5g-tdT* mice, were used for ex vivo Ca^2+^ imaging, immunofluorescence studies and fluorescence recovery after photobleaching (FRAP) assays [15,34].

Adipocyte-specific Cx43 knockout mice were generated by breeding B6;*129s7-Gja ^Tm1Dlg^*/J mice (Jackson Laboratory, Stock No. 008039; RRID: IMSR_JAX:008039) with B6.*FVB-Tg(Adipoq-cre)1Evdr*/J mice (Jackson Labotatory, Stock No. 028020; RRID: IMSR_JAX:028020). Offspring, referred to as Adipo^∆Cx43^ KO, were used for experiments. Cre-negative littermates served as controls in immunofluorescence studies and vascular contractility assays.

All mouse genotypes were verified by Transnetyx (Boston, MA, USA). Mice were housed in Optimice cages from Animal Care Systems with bedding from Frontier Distributing, cotton and paper nestlets (NES3600) from Ancare (Bellmore, NY, USA), and red mouse igloos (K3327) from Bio-Serve (Flemington, NJ, USA) in a temperature-controlled environment on a 12 h:12 h light/dark cycle with ad libitum access to food (Teklad Irradiated Global 19% Protein Extruded Rodent Diet, 2919) from Inotiv (Lafayette, IN, USA) and distilled tap water. All works involving animals were conducted following the standards established by the National Institutes of Health (NIH) Guide for the Care and Use of Laboratory Animals, and were approved by the Institutional Animal Care and Use Committee (IACUC) at Michigan State University (AUF# PROTO020400255). All animals were randomly assigned to experimental groups based on age and genotype prior to the initiation of experimental procedures.

### 4.2. Drugs and Reagents

(±) Norepinephrine (+) bitartrate salt (Sigma, St Louis, MO, USA, A7256, NE) was focally applied to samples of PVAT during Ca^2+^ imaging experiments using pressure ejection from a glass micropipette and bath applied to vessels in organ baths during wire myography experiments. Carbenoxolone disodium salt (Sigma, C4790, Cbx) was added to imaging chamber baths to achieve a final concentration of 50 µmol/L in modified Krebs buffer during Ca^2+^ imaging experiments and FRAP experiment. 43Gap26 (Anaspec Inc., Fremont, CA, USA, 62644) was prepared in modified Krebs buffer and applied at 100 µmol/L during Ca^2+^ imaging experiments. Stock solutions of Calcein acetoxymethylester (Invitrogen, Waltham, MA, USA, C3100MP, Calcein AM) were prepared in dimethyl sulfoxide (DMSO) and diluted to a final concentration of 4 µmol/L in modified Krebs buffer during FRAP experiments.

Modified Krebs buffer was used for Ca^2+^ imaging and FRAP experiments and contained (in mmol/L) 121 NaCl (Sigma, 746398), 5.9 KCl (Sigma, 74636), 2.5 CaCl_2_ (Sigma, C1016), 1.2 MgCl_2_ (Thermo Fisher Scientific, Pittsburgh, PA, USA, 3818), 1.2 NaH_2_PO_4_ (Thermo Fisher Scientific, 2444-01), 10 HEPES (Sigma, H3375), 21.2 NaHCO_3_ (Thermo Fisher Scientific, S233), 1 Sodium pyruvate (Sigma, P5280), 8 glucose (Sigma, G7021). Phosphate-buffered saline (PBS) was used in immunofluorescence studies and contained (in mol/L) 0.137 NaCl, 2.7 KCl, 10 Na_2_HPO_4_ (Thermo Fisher Scientific, 3828-01), 0.0018 KH_2_PO_4_ (Thermo Fisher Scientific, 3246-01) and 0.02% Sodium azide (Sigma, S2002). DMEM (11039021) and ultrapure water (SH30538.03) was sourced from Thermo Fisher Scientific.

In vessel contractility experiments, a low Ca^2+^ solution dissection solution used while preparing samples that contained (in mmol/L) 140 NaCl, 5 KCl, 0 CaCl_2_, 1 MgCl_2_, 10 HEPES, 10 glucose, 0.01 Sodium nitroprusside (Sigma, 71780), 0.01 Diltiazem (Sigma, D2521) and 1 mg/mL albumin (Thermo Fisher Scientific, J10856-09). During experiments, samples were incubated in buffer that contained (in mmol/L) 135 NaCl, 4 KCl, 1.8 CaCl_2_, 1 MgCl_2_, 10 HEPES, 10 glucose. Samples were stimulated with a high potassium 60 mM K solution that contained (in mmol/L) 75 NaCl, 60 KCl, 1.8 CaCl_2_, 1 MgCl_2_, 10 HEPES, 10 glucose. The pH of all solutions was adjusted to 7.4 with NaOH.

### 4.3. Tissue Collection

Aortic and mesenteric PVAT (aPVAT, mPVAT) and white adipose tissue (WAT) were collected from male and female *Adipoq^cre^;GCaMP5g-tdT* mice. Following anesthesia and cervical dislocation, the abdominal cavity was opened to access mPVAT and perigonadal WAT and the rib cage was opened to access aPVAT. All tissues were kept in ice-cold DMEM-F12.

For Ca^2+^ imaging, and FRAP experiments, small sections of mPVAT, aPVAT, and WAT were excised and secured with pins (Thermo Fisher Scientific, 26002-20) in Sylgard (Dow-Corning, Midland, MI, USA)-coated custom rectangular imaging dishes (imaging chamber, 2.1 mm × 1.5 cm) in ice-cold modified Krebs buffer.

For vascular contractility assays, the entire mesentery was removed from male and female Cx43 knockout and control wild type mice. The mesentery was pinned out to facilitate access to the individual vessel branches in ice-cold dissection buffer. Resistance mesenteric arteries with or without mPVAT were carefully isolated from the second- and third-order branches of the mesenteric arterial network in ice-cold dissection buffer [46,61].

### 4.4. Calcium Imaging

Samples of adipose tissues were visualized using a ×20 widefield water-immersion objective lens (1.0 numerical aperture, XLUMPlanFLN20xW, Olympus, Tokyo, Japan) mounted on an upright BX51WI fixed-stage microscope (Olympus, Tokyo, Japan). Fluorescence illumination was provided by a Lumencor AURA light engine (Lumencor, Beaverton, OR, USA). The excitation light for GCaMP5g was filtered through a 485/20 nm band-pass filter, and emitted fluorescence signal was collected through a 515 nm long-pass filter. The tdTomato signal was excited using light filtered through a 535/20 nm band-pass filter, and emitted fluorescence signal was collected through a 610/75 nm band-pass emission filter before detection. Imaging data were recorded every second using a Photometrics Prime BSI camera (Teledyne, ON, Canada) and NIS-Elements AR 6.02.03 64-bit (Nikon, Tokyo, Japan). Videos and images were saved as .nd2 files for further analysis using FIJI (v.2.14.011.54, National Institutes of Health). Throughout the experiment, 1× modified Krebs buffer was perfused at a rate of 2–4 mL/min (AutoMate Scientific, Berkeley, CA, USA) and maintained at 37 °C with an inline heater (TC-344C, Warner Instruments, Hamden, CT, USA) [15,34].

NE was focally applied to adipocytes using a glass micropipette constructed using a P-87 Flaming-Brown Micropipette Puller (Sutter Instruments, Novato, CA, USA), and backfilled with NE dissolved in modified Krebs buffer. NE was delivered using gentle positive pressure from a 1 mL syringe connected to a pipette holder. This method enabled delivery of small volumes of NE to an isolated patch of adipocytes. Antagonists were bath applied and samples were preincubated for 10 min with either Cbx (50 µmol/L) or 43Gap26 (100 µmol/L) before NE application [15,62,63,64].

Fluorescence changes were quantified as ΔF/F_0_ using the equation ΔF/F_0_ = (F(t) − F_0_)/F_0_, where F(t) is the fluorescence intensity at each time point (t), and F_0_ is the baseline fluorescence, defined as the average intensity over the first 20 frames. For each video, the F_0_ image was calculated, the F_0_ was subtracted from each frame, which is divided by F_0_ at each time point. Peak responses were determined for each responding cell in Excel and plotted as scatter plots in Prism.

### 4.5. Immunofluorescence Staining and Imaging

Immunofluorescence labeling was imaged using a Zeiss LSM 880 NLO system (Carl Zeiss, Oberkochen, Germany) equipped with 594 and 488 nm laser lines. Images were collected with a Plan-Apochromat ×20 objective (NA = 0.8). Acquisition parameters (photomultiplier gain, laser intensity, pin hole size) were optimized using positive samples (i.e., those exposed to both primary and secondary antibodies), across all tissue types to confirm that the fluorescence emission did not saturate the detectors, or produce autofluorescence in the negative control samples. After optimization, the same settings were applied to all samples. Image stacks (approximately 100, three channels, 1024 × 1024 8-bit images, 1 μm step-size) were acquired using sequential line scanning to minimize channel crosstalk. All images were saved in .czi format for analysis in FIJI.

For all incubation and wash steps, tissues were continuously agitated on an orbital shaker at room temperature. Samples were fixed in 4% paraformaldehyde for 2 h, then washed three times for 10 min in PBS. Tissues were blocked for 1 h in blocking buffer [10% normal donkey serum (Jackson Immuno Research, West Grove, PA, USA, 017-000-121), 1% bovine serum albumin (Jackson Immuno Research, 001-000-162, BSA), 0.4% Triton X-100 (Sigma, 93443); in PBS] and then incubated for 3 days in the following primary antibodies: connexin-43 (Sigma, C6219, 1:500), Caveolin-1 (Santa Cruz, Dallas, TX, USA, sc-53564, 1:100). After incubation in the primary antibody, samples were washed 3 times for 1 h in PBS and incubated with the secondary antibodies for 1 day (1:500 dilution; donkey anti-mouse IgG Alexa 488; donkey anti-rabbit IgG Alexa 594, Jackson Immuno Research). Samples were counterstained with 4′,6-diamidino-2-phenylindole dihydrochloride (DAPI, Sigma, D8417, 1:10,000) in PBS for 1 h. Following two additional 1 h rinses in PBS, samples were mounted on slides (VWR, 16004-370) using Coverwell Imaging Chambers (Electron Microscopy Science, Hatfield, PA, USA, 70327-05) and Hydromount (National Diagnostics, Atlanta, GA, USA, H5-106). Mounted slides were stored in the dark at 4 °C before imaging.

### 4.6. Fluorescence Recovery After Photobleaching

FRAP experiments were performed on a Nikon AXR Confocal system (Nikon Instruments Inc., Melville, NY, USA). Imaging was performed through the ApoLWD 25 × 1.10 WDIC N2 water-immersion objective (numerical aperture, NA = 1.1), providing high resolution and optimal light collection for confocal imaging (resolution 1024 × 1024 with line averaging of 2). Both photobleaching and fluorescence imaging of Calcein AM were performed using an argon-ion laser (488 nm) and a photomultiplier tube for detection of fluorescence in the 500 and 550 nm range. Acquisition was performed at speed 9 (pixel dwell 4 µs), averaging number 1, and 8-bit depth. Photobleaching was carried out at scan speed 3 (pixel dwell 50.42 µs), 50 scans, and 4 iterations.

The adipocytes were loaded with 4 µmol/L of Calcein AM for 15 min and then washed three times with modified Krebs buffer (1 min each) before being subjected to the FRAP method. Gap junction communication in adipocytes was blocked by incubation with 100 µmol/L of Cbx for 15 min. Cbx was present in the modified Krebs buffer during the entire FRAP experiment. To perform precise measurements, three regions of interest (ROIs) were selected. An ROI was manually selected around a cell for photobleaching, a reference ROI within an unbleached area was drawn to correct for fluorescence changes associated with acquisition bleaching, and an ROI was placed outside the cells to measure the background. Prior to photobleaching, initial images were recorded to determine the initial fluorescence intensity. The selected ROI was photobleached at 100% power and time-lapse imaging was then recorded to monitor Calcein AM recovery every 3 s for up to 3 min. The fluorescence intensity of the bleached ROI was normalized to the reference ROI at each time point. Normalized values were used to calculate the percentage mobile fraction of fluorescence using the equation: mobile fraction percentage = [(F_FR_ − F_B_)/(F_I_ − F_B_)] × 100, where F_FR_ is the fluorescence intensity in the bleached region after full recovery, F_B_ after bleaching, and F_I_ before bleaching. The image and fluorescence recovery data were exported from Nikon NIS-Elements AR 6.10.01 64-bit to GraphPad Prism (10. 6.1) (GraphPad Software, San Diego, CA, USA) for plotting. Experiments showing considerable fluctuations in fluorescence intensity within reference ROI or base ROI were excluded from the analysis [41,42,43,44,65].

### 4.7. Vascular Contractility

Small mesenteric artery segments with or without PVAT were isolated from the second- and third-order branches of the mesenteric arterial network and mounted on two 40 µm wires in a DMT Multi-Wire Myograph System, Model 620 M (Danish Myo Technology A/S, Hinnerup, Denmark) [65]. Data were recorded using a PowerLab Data Acquisition unit (ADInstruments, Colorado Springs, CO, USA). The vessels were gassed with clinical blood gas (5% carbon dioxide/21% oxygen/balanced nitrogen) and maintained at 37 °C for 1 h, with washes every 20 min. Wall tension and diameter were then normalized using a standardized procedure. After normalization, the vessels were washed two times for 20 min to achieve a stable baseline. A total of 60 mmol/L high potassium PSS (KPSS) was applied in the chamber for 5 min to establish viability of the vessel and initial maximum contraction. After returning to baseline in PSS for 40 min with washes every 20 min, cumulative concentration–response curves to NE were conducted with 3 min intervals for the vessels without PVAT and 6 min intervals for the vessels with PVAT. Percentage loss of NE-induced tone and potencies (EC_50_) were calculated for WT and Cx43 KO mesenteric artery [14,46,61,66].

### 4.8. Real-Time Quantitative Polymerase Chain Reaction

Total mRNA was extracted from frozen samples of mesenteric PVAT. Samples were mechanically lysed using mechanical dissociation (Bead Bug, Stellar Scientific, Baltimore, MD, USA) in lysing Matrix D (MPBio, Irvine, CA, USA). RNA was isolated using miRNAeasy Mini Kit (QGN-217004, Qiagen, Hilden, Germany) and quantified on Nanodrop 1000 spectrophotometer (Thermo Fisher). Purified RNA was stored at −80 °C. cDNA was synthesized from 0.3 µg RNA using Verso cDNA synthesis kit (Thermo Fisher Scientific, AB1453A) and the MiniAmp Thermal Cycler (Thermo Fisher Scientific) with the following parameters: 42 °C for 30 min, 95 °C for 2 min, and held at 4 °C.

Quantitative gene expression analysis was performed on QuantStudio 3 Real-Time PCR system (Applied Biosystems, Carlsbad, CA, USA) with PowerTrack^TM^ SYBR Green Master Mix for qPCR (Applied Biosystems, Carlsbad, CA, USA, A46109): 95 °C for 10 min, 40 cycles of 95 °C for 10 s and 60 °C for 1 min. Relative gene expressions for Adipo^∆Cx43^ KO and WT mice were normalized to the respective housekeeping gene (RPS6) and were analyzed using the 2^−ΔΔCt^ method. Table 1 shows PCR primers sequences and related key resources.

### 4.9. Statistical Analysis

All experiments were repeated at least three independent times in separate mice. Statistical analyses were performed using two-tailed unpaired Student’s *t*-test or one-way ANOVA in GraphPad Prism software (10.6.1). As no sex-dependent differences were observed, data from males and females were combined for analysis. Results are expressed as mean ± standard error of the mean (SEM) and a *p*-value lower than 0.05 was considered statistically significant.

## Figures and Tables

**Table 1:** Sequences of primers for real-time PCR analysis. Primers were validated by melt curve analysis and serial dilution standard curve (5-point, 5-fold dilution).

Genes	Primer Sequence		Product Size
Cx26	Forward	5′-GATGTTGGCCTTTGGGTTATG-3′	211
	Reverse	5′-CGGCATATCCTATCTGTCTCTTAC-3′	
Cx32	Forward	5′-CTATGGTCCCTGCAGCTTATC-3′	151
	Reverse	5′-GCACCTTGTGTCTCTTTACCT-3′	
Cx37	Forward	5′-CACTGGCTGCTTACCAGAAT-3′	87
	Reverse	5′-CGAGGGTTCACAGAACACTTAG-3′	
Cx40	Forward	5′-GCACCAGATACCGAGATTTAC-3′	154
	Reverse	5′-GGCTCTTCTTCACCATTCTATC-3′	
Cx43	Forward	5′-ACAGCGGTTGAGTCAGCTTG-3′	106
	Reverse	5′-GAGAGATGGGGAAGGACTTGT-3′	
Cx45	Forward	5′-GTGAACAGGGCAAACCAATTC-3′	153
	Reverse	5′-GACTCTCCTCCTACAGCAGTTA-3′	
Cx46	Forward	5′-GCGGGCCAGTACTTTCTATAC-3′	232
	Reverse	5′-CATCTGGGTTGAAGTGGTTAGT-3′	
Cx50	Forward	5′-CTTATGCCACTCCATCCTCTTC-3′	209
	Reverse	5′-TCCCTGTCTCGTCTCTCATAAT-3′	
RPS6	Forward	5′-GAAGCGCAAGTCTGTTCGTG-3′	237
	Reverse	5′-GTCCTGGGCTTCTTACCTTCT-3′	

**Table 2:** Relative expression of connexin mRNA in mPVAT.

Gene	Group	∆Ct	∆∆Ct	Fold Change
Cx26	WT	4.49 ± 0.84	0.00 ± 0.84	1.13 ± 0.51
	Adipo^∆Cx43^	3.54 ± 0.68	−0.95 ± 0.68 *	2.14 ± 1.06
Cx32	WT	6.01 ±1.14	0.00 ± 1.14	1.26 ± 0.87
	Adipo^∆Cx43^	5.44 ± 0.54	−0.57 ± 0.54	1.57 ±0.51
Cx37	WT	5.56 ± 0.48	0.00 ± 0.48	1.04 ± 0.32
	Adipo^∆Cx43^	5.32 ± 0.60	−0.24 ± 0.60	1.27 ± 0.50
Cx40	WT	4.65 ± 0.66	0.00 ±0.66	1.08 ± 0.40
	Adipo^∆Cx43^	3.97 ± 0.48	−0.68 ± 0.48	1.68 ±0.57
Cx43	WT	4.18 ± 0.48	0.00 ± 0.48	1.04 ± 0.31
	Adipo^∆Cx43^	4.17 ± 0.61	−0.01 ± 0.61	1.09 ± 0.44
Cx45	WT	4.19 ± 0.55	0.00 ± 0.55	1.06 ± 0.40
	Adipo^∆Cx43^	3.75 ± 0.41	−0.44 ± 0.41	1.40 ± 0.39
Cx46	WT	5.84 ± 1.16	0.00 ± 1.16	1.25 ± 0.75
	Adipo^∆Cx43^	5.05 ± 0.55	−0.79 ± 0.55	1.85 ± 0.82
Cx50	WT	3.86 ± 1.18	0.00 ± 1.18	1.23 ± 0.67
	Adipo^∆Cx43^	3.25 ± 0.59	−0.61 ± 0.59	1.63 ± 0.59

Genes quantified by Real-Time qPCR in mPVAT from Adipo^∆Cx43^ KO and WT mice. (n = 6–8 animals per experimental group, data are presented as mean ± SEM, * *p* = 0.048, two-tailed *t* test). All RT-qPCR reactions were performed in triplet using PowerTrack SYBR Green master mix. Ct values were analyzed using the ∆∆Ct method.

## Data Availability

The original contributions presented in this study are included in the article and data are available upon reasonable request to the corresponding author.

## Pre-Print Server

The manuscript has not been shared as a preprint.

## Synopsis

This study shows that adipocytes in perivascular adipose tissue are gap junction-coupled and that these gap junctions, which may be composed of connexin-43, play an important role in distributing signals initiated by sensing norepinephrine. These mechanisms could participate in regulating how perivascular adipose tissue influences blood vessel contractility and, hence, susceptibility to diseases such as hypertension.

## Abbreviations

PVAT, perivascular adipose tissue; mPVAT, mesenteric perivascular adipose tissue; aPVAT, aortic perivascular adipose tissue; WAT, white adipose tissue; Cx43, connexin-43; Ca^2+^, calcium; NE, norepinephrine; Cbx, carbenoxolone; FRAP, fluorescence recovery after photobleaching.
